# Urban Wetland Sediments in Yangzhou: Physicochemical Properties, Microbial Communities, and Functional Associations

**DOI:** 10.3390/microorganisms13081843

**Published:** 2025-08-07

**Authors:** Dongmei He, Liwen Li, Runyang Zhou, Sumei Qiu, Wei Xing, Yingdan Yuan

**Affiliations:** 1 Jiangsu Academy of Forestry, Nanjing 211153, China; 2 Yancheng Coastal Wetland Ecosystem Positioning Observation Station in Jiangsu Province, Yancheng 224136, China; 3 College of Horticulture and Landscape Architecture, Yangzhou University, Yangzhou 225009, China

**Keywords:** urban wetland sediments, microbial community, sediment carbon, sediment nitrogen, NCM

## Abstract

Urban wetlands play a crucial role in maintaining ecological balance, carbon sequestration, and water purification. Sediments are key carriers for wetlands to store elements such as carbon, nitrogen, and phosphorus in the aquatic environment. This study analyzed different sediment layers of seven wetlands in Yangzhou, aiming to explore the relationship between physicochemical factors and microbial communities in wetland sediments, as well as to predict the functions of microbial communities. Functional prediction of microbial communities was conducted based on amplicon sequencing analysis, and the neutral community model was used to determine the formation and evolution process of microbial communities. The results showed that in three wetlands, namely Zhuyu Bay (ZYW), Luyang Lake (LYH), and Runyang Wetland (RYSD), the contents of carbon components (total carbon, total soluble carbon, microbial biomass carbon) in the 0–20 cm sediment layer were higher, while the carbon component contents in Baoying Lake (BYH) showed the opposite trend. Among them, the contents of total nitrogen, alkali-hydrolyzable nitrogen, total phosphorus, available phosphorus, total potassium, and available potassium in the 0–20 cm sediment layer of Runyang Wetland (RYSD) were significantly the highest. This indicates that in Runyang Wetland (RYSD), the 0–20 cm layer has more abundant carbon components and mineral nutrients compared to the 20–40 cm layer. Among the seven wetlands, it was found that the content of total potassium was all greater than 10 g/kg, which was much higher than the contents of total phosphorus and total nitrogen. Analysis of microbial communities revealed that the dominant archaeal phyla were Thaumarchaeota and Euryarchaeota, and the dominant bacterial phyla were Proteobacteria and Acidobacteria. The distribution of functional genes was mainly concentrated in Zhuyu Bay (ZYW) and Luyang Lake (LYH). Zhuyu Bay Wetland (ZYW) had potential advantages in light utilization function, and Luyang Lake (LYH) had potential advantages in carbon and nitrogen cycle functions. The assembly process of the archaeal community was mainly affected by stochastic processes, while the bacterial community was mainly affected by deterministic processes. However, water content, total phosphorus, and available potassium all had strong correlations with both archaeal and bacterial communities. The research results preliminarily reveal the connections between the physicochemical properties of sediments, microbial communities, and their potential functions in Yangzhou urban wetlands, providing an important scientific basis for the protection and management of wetland ecosystems.

## 1. Introduction

Wetland sediments, serving as a transitional zone between terrestrial and aquatic ecosystems, are rich in nutrients like carbon, nitrogen, and phosphorus [1]. They function not only as a storage repository for large amounts of carbon and nitrogen [2,3], but also as an active exchange platform for these elements. Studies have shown that the organic matter content in wetland sediments is typically higher than that in agricultural and forest ecosystems [4]. During the sedimentation process, this organic matter is transformed into carbon through microbial action and is stored long-term in the sediments [5]. Even minor changes in the carbon content of wetland ecosystems can potentially trigger climate changes in surrounding regions and even on a global scale [6].

One of the key factors influencing the formation of wetland sediments is sedimentary microorganisms. These microorganisms are highly diverse and can be broadly categorized into bacteria [7], fungi, and archaea, with bacteria having the highest species richness, followed by archaea, and then fungi [8]. Xu et al., through metagenomic analysis of the microbial community in the Pahai Plateau, demonstrated that the primary carbon fixation pathways in wetland microorganisms are the Calvin cycle, reductive tricarboxylic acid cycle, and 3-hydroxypropionic acid cycle. The main carbon-fixing microbial groups are the Proteobacteria, Chloroflexi, and Crenarchaeota, while the primary nitrogen-fixing microbial groups include Proteobacteria, Nitrospirae, Verrucomicrobia, Actinobacteria, Thermodesulfobacteria, and Firmicutes [9]. Cheung et al. also found that archaea play a significant role in methane (CH4) and carbon cycling [8]. It is evident that sedimentary microorganisms have a substantial impact on the composition of wetland sediments.

Variations in sedimentary microbial communities can influence the overall structure and functional changes in wetland ecosystems [10]. As a crucial component of wetland ecosystems, microorganisms play a significant role in biogeochemical cycling [11]. Establishing the associations between microbial communities and physical, chemical, and biological factors can provide deeper insights into the drivers of microbial community formation. This is of vital importance for the restoration of wetland ecosystem functions [12]. Meanwhile, numerous studies have demonstrated a strong correlation between microbial communities and environmental factors [13,14]. Therefore, exploring the factors influencing microbial communities in wetland sediments is of great significance for understanding the potential functions of ecosystems.

Microorganisms in wetland sediments play a key role in stabilizing the Yangzhou wetland ecosystem. Through their transformation of carbon, nitrogen, and other substances and their ability to decompose pollutants [15], they ensure the relative stability of soil composition and physicochemical properties, vegetation structure, and greenhouse gas concentrations in the atmosphere within the region [16,17,18]. Alpine and coastal wetlands now receive much attention [19,20], but plain wetland sediment research is relatively insufficient. Plain wetlands typically have freshwater, subtropical climates, rich aquatic vegetation and migratory bird habitats; coastal ones lie in land–sea transition zones, with tides, high salinity and marine life aggregation, and it is susceptible to abiotic interference [21]; alpine ones feature high altitude, cold climates, snowmelt recharge and cold-tolerant species [22]. Thus, studying sediments and material cycles in Yangzhou’s plain wetland is of great practical and scientific value.

Here, we mainly focused on three questions: (1) investigating the physicochemical properties and microbial community composition of different sediment layers (0–20 cm and 20–40 cm) in Yangzhou urban wetlands; (2) identifying the factors influencing microbial communities in different sediment layers (0–20 cm and 20–40 cm) of Yangzhou urban wetlands; and (3) exploring the potential functions of microbial communities in sediments from different wetlands in the Yangzhou area.

## 2. Materials and Methods

### 2.1. Sediment Collection

**Figure 1:**
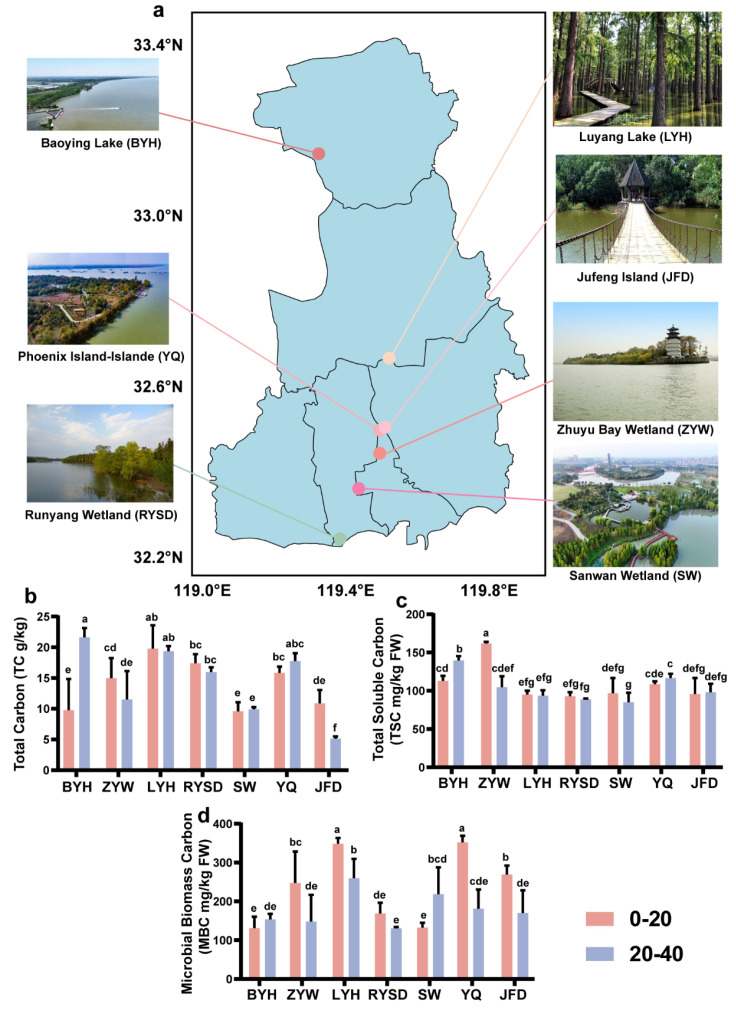
Sediment sampling site map and differences in carbon fractions. (**a**) The geographical locations of seven urban wetlands within Yangzhou City. (**b**) Total Carbon (TC); (**c**) Total Soluble Carbon (TSC); (**d**) Microbial Biomass Carbon (MBC). Baoying Lake (BYH), Zhuyu Bay (ZYW), Luyang Lake (LYH), Runyang Wetland (RYSD), Sanwan (SW), Phoenix Island-Islande (YQ), and Jufeng Island (JFD). Values are presented as mean ± standard error (n = 3). Lowercase letters represent the differences among different wetlands (*p* < 0.05).

The sediment sampling sites are in Yangzhou City, Jiangsu Province, China. Yangzhou is situated at the confluence of the Yangtze River and the Grand Canal, characterized by a dense network of rivers and lakes, and abundant wetland resources. We selected seven types of wetlands within Yangzhou, namely Baoying Lake (BYH), Zhuyu Bay (ZYW), Luyang Lake (LYH), Runyang Wetland (RYSD), Sanwan (SW), Phoenix Island-Islande (YQ), and Jufeng Island (JFD). The distribution of these seven wetlands in Yangzhou is shown in Figure 1. In July, three biological replicates were set up for each sediment layer in each wetland, and sediments from the 0–20 cm and 20–40 cm layers of each wetland were collected using a piston corer. We removed stones, debris, and other impurities from the sediments. Subsequently, all sediments were packaged separately into sterile, sealed bags labeled with corresponding site numbers. The samples were then transported to the laboratory in a cooler. The collected sediments were divided into three portions: one portion was stored at 4 °C for the measurement of total carbon content, soil microbial biomass carbon, and dissolved total carbon content; another portion was stored at −80 °C for the analysis of sediment microbial communities; and the remaining portion was air-dried at room temperature and then used for the determination of other physicochemical properties.

### 2.2. Determination of Physicochemical Properties of Sediments

The pH of the sediment was measured using a pH meter. The water content (WC) was determined by the gravimetric method. Total nitrogen (TN) was measured by the Kjeldahl method following sulfuric acid digestion. Ammonium nitrogen (AN) was determined by the alkaline diffusion method. Total phosphorus (TP) was measured by the NaOH fusion–molybdenum-antimony resistance spectrophotometry method. Available phosphorus (AK) in the soil was determined by the sodium bicarbonate extraction–molybdenum-antimony resistance colorimetric method. Total potassium (TK) was measured by the NaOH fusion–flame photometry method. Available potassium (AK) was determined by the ammonium acetate extraction–flame photometry method [23]. Microbial biomass carbon (MBC) in the sediment was extracted by the chloroform fumigation–K_2_SO_4_ extraction method and measured using a carbon–nitrogen analyzer. Soluble total carbon (TSC) was determined by high-speed centrifugation followed by analysis using a carbon–nitrogen analyzer [24]. Total carbon (TC) was measured by combustion–infrared absorption spectroscopy.

### 2.3. Sediment Microbial Analysis

Total genomic DNA was extracted from the two sediment layers of seven wetlands using the CTAB and SDS methods [25]. The concentration and purity of the extracted DNA samples were assessed on a 1% agarose gel, and the DNA was diluted to 1 ng·μL^−1^ with sterile water according to the concentration. Bacterial 16S rRNA gene V3-V4 region libraries were amplified using specific primers 338F (5′-ACTCCTACGGGAGGCAGCAG-3′) and 806R (5′-GGACTACHVGGGTWTCTAAT-3′), while the V4-V5 hypervariable region of archaeal 16S rDNA was amplified using specific primers 519F (5′-CAGCCGCCGCGGTAA-3′) and 915R (5′-GTGCTCCCCCGCCAATTCCT-3′). The amplified DNA libraries were indexed using the TruSeq^®^ DNA PCR-Free Sample Preparation Kit (Illumina, San Diego, CA, USA) and assessed for quality on a Qubit^®^ 2.0 fluorometer (Thermo Scientific, Waltham, MA, USA) and an Agilent Bioanalyzer 2100 system. After purification and quantification of the libraries, high-throughput sequencing was conducted on the Illumina NovaSeq 6000 platform (Personalbio Company, Shanghai, China) in a 2 × 250 bp paired-end mode following standard operating procedures. The processing of sequencing raw data included quality control using QIIME (version 2, accessed on 1 September 2024) and merging of paired-end reads using FLASH (version 1.2.7, accessed on 1 September 2024) [26,27]. For annotation of bacterial and archaeal sequences, the UCHIME algorithm (UCHIME, URL: http://www.drive5.com/usearch/manual/uchime_algo.html, accessed on 1 September 2024) was used to align sequences with the Silva database [28,29].

### 2.4. Neutral Community Model

The Neutral Community Model (NCM) is a fundamental theoretical framework for understanding microbial community structure. Its core premise emphasizes that stochastic processes, including ecological drift, and migration–diffusion are primary drivers of community assembly. The model operates by analyzing observational data on microbial relative abundance and occurrence frequency, using quantitative methods to discern assembly patterns. The coefficient of determination (*R*^2^) is a key parameter for assessing NCM fit. Higher *R*^2^ values indicate greater congruence with neutral model predictions, reflecting stronger stochastic influence and weaker deterministic forces, such as environmental filtering and biotic interactions. Migration–diffusion quantity (*Nm* = *N* × *m*) quantifies species dispersal potential; elevated *Nm* values are associated with more uniform species distributions across communities. Based on the OTU sequence data obtained from microbial analysis, after data normalization processing, this study constructed a neutral community model using the Hmisc package in R language, aiming to systematically quantify the role of stochasticity in community assembly from the perspective of community ecology [30]. Among them, the migration rate (*Nm*) is calculated as the product of the migration parameter m, which is fitted using the minpack.lm package, and the community size N; the coefficient of determination (*R*^2^) is calculated as the ratio of the sum of squared residuals to the total sum of squared deviations, and is used to reflect the fitting effect of the model.

### 2.5. Data Analysis

In this study, one-way analysis of variance (ANOVA) and least significant difference (LSD) test (*p* < 0.05) were used for statistical analysis of physical and chemical factors. For microbial community analysis, first, the vsearch tool in QIIME 2 software was used to process the sequences. After rarefaction treatment (Sparsification depth see Figure S1), the relevant indices of α diversity and β diversity were calculated, and visualization was realized using R language. To further analyze the microbial community structure, the R package edgeR (version 4.0.16) was used for differential abundance analysis [31]; the R package igraph (version 2.0.3) was used to construct co-occurrence networks, analyze their topological structures, and complete visualization to clarify the interaction patterns between species; based on distance thresholds of 1.1, 1.5, and 1.3, the nodes and edges of bipartite networks were calculated, and bipartite network results were obtained to show the shared phyla of microbial communities in different samples. After normalizing the OTU data obtained from microbial analysis, the R package linkET (version 0.0.7.4) was used to analyze the correlation between physical and chemical factors and microorganisms; finally, the R package microeco (version 1.8.0) was used to predict the ecological functions of wetland microorganisms.

## 3. Results

### 3.1. Analysis of the Physicochemical Properties of Urban Wetland Sediments

We determined the carbon fractions in sediments from seven wetlands (Figure 1a–d). The results showed that in the 0–40 cm sediment layer, LYH had the highest total carbon (TC) and microbial biomass carbon (MBC) contents, with average values of 19.57 g/kg and 304.01 mg/kg, respectively. The wetland with the highest total soluble carbon (TSC) content was ZYW, with an average value as high as 133.25 mg/kg. A comparison of carbon fraction contents across different sediment layers revealed that in BYH, the carbon fraction contents in the 0–20 cm sediment layer were all lower than those in the 20–40 cm layer, while in the three wetlands ZYW, LYH, and RYSD, the carbon fraction contents were higher in the 20–40 cm sediment layer. This indicates that BYH has more abundant carbon fractions in the 0–20 cm sediment layer, whereas ZYW, LYH, and RYSD have more abundant carbon fractions in the 20–40 cm sediment layer. Further analysis of MBC in sediments from different wetlands showed that only BYH and SW had the lowest MBC contents in the 0–20 cm sediment layer, while the other five wetlands had the highest MBC contents in the 0–20 cm layer. This phenomenon may be related to the composition of microbial communities.

**Figure 2:**
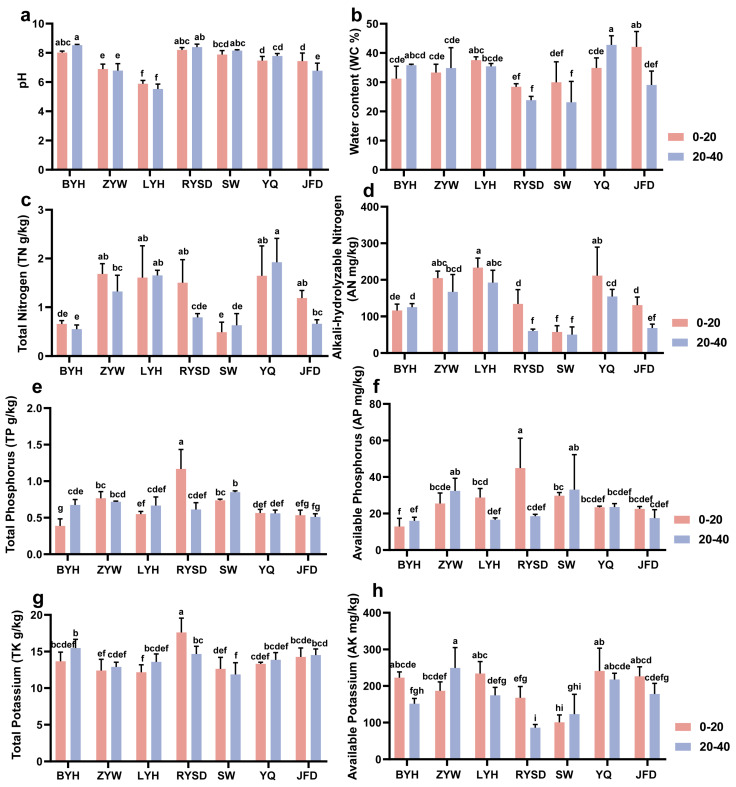
Differences in physical and chemical properties of sediments in different wetlands. (**a**) pH; (**b**) Water content (WC); (**c**) Total Nitrogen (TN); (**d**) Alkali-hydrolyzable Nitrogen (AN); (**e**) Total Phosphorus (TP); (**f**) Available Phosphorus (AP); (**g**) Total Potassium (TK); (**h**) Available Potassium (AK). Baoying Lake (BYH), Zhuyu Bay (ZYW), Luyang Lake (LYH), Runyang Wetland (RYSD), Sanwan (SW), Phoenix Island-Islande (YQ), and Jufeng Island (JFD). Values are presented as mean ± standard error (n = 3). Lowercase letters represent the differences among different wetlands (*p* < 0.05).

We measured the pH of sediments from the seven wetlands and found that the pH of LYH was less than 6, significantly lower than that of sediments from other wetlands. We also found that only the JFD wetland showed a significant difference in pH between different sediment layers (Figure 2a). In terms of water content, the RYSD and SW wetlands exhibited similar characteristics. In the 0–20 cm sediment layer, the water content of both was approximately 30%, and there was still no significant difference in water content in the 20–40 cm layer, but both were lower than those of the other five wetlands (Figure 2b). In addition, we determined the relevant physical and chemical factors of nitrogen, phosphorus, and potassium in the sediments (TN, AN, TP, AP, TK, AK) (Figure 2c–h). It was found that the contents of the six physical and chemical properties in RYSD varied significantly between different sediment layers, with the highest amount of significantly occurring in the 0–20 cm layer. In the 0–20 cm sediment layer, the wetlands with the highest contents of the six physical and chemical factors were as follows: ZYW for TN (1.69 ± 0.12 g/kg), LYH for AN (233.52 ± 15.09 mg/kg), RYSD for TP (1.17 ± 0.15 g/kg), AP (44.91 ± 9.42 mg/kg), and TK (17.63 ± 1.12 g/kg), and YQ for AK (241.32 ± 35.90 mg/kg). In the 20–40 cm sediment layer, the wetlands with the highest contents of the six physical and chemical factors were as follows: YQ for TN (1.92 ± 0.28 g/kg), LYH for AN (192.60 ± 19.42 mg/kg), SW for TP (0.85 ± 0.01 g/kg) and AP (33.10 ± 11.06 mg/kg), BYH for TK (15.48 ± 0.68 g/kg), and ZYW for AK (249.56 ± 32.12 mg/kg). Further research found that in the sediments of the seven wetlands, the content of TK was all greater than 10 g/kg, far higher than the contents of TN and TP; the content of AK was also much higher than that of AP.

### 3.2. Analysis of Archaeal and Bacterial Communities in Urban Wetland Sediments

**Figure 3:**
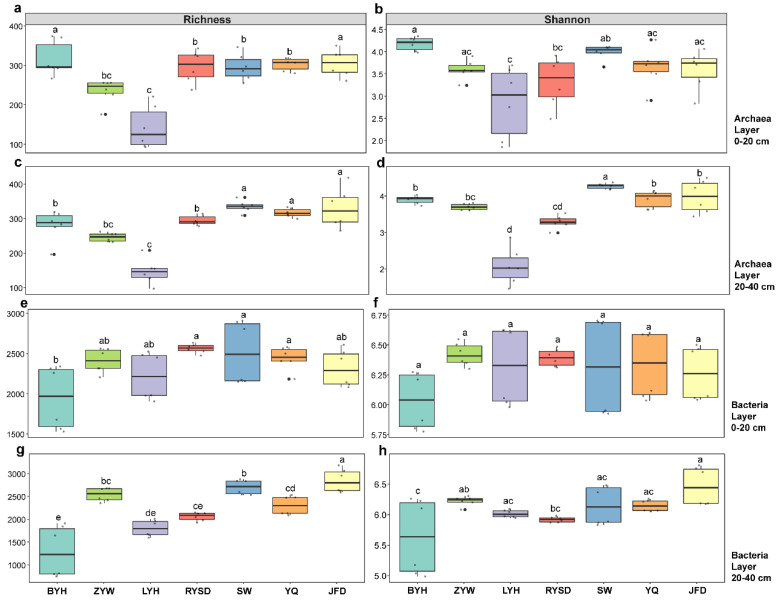
Analysis of Sediment Microbial α-Diversity. (**a**) Archaeal richness in the 0–20 cm sediment layer; (**b**) Archaeal Shannon index in the 0–20 cm sediment layer; (**c**) Archaeal richness in the 20–40 cm sediment layer; (**d**) Archaeal Shannon index in the 20–40 cm sediment layer; (**e**) Bacterial richness in the 0–20 cm sediment layer; (**f**) Bacterial Shannon index in the 0–20 cm sediment layer; (**g**) Bacterial richness in the 20–40 cm sediment layer; (**h**) Bacterial Shannon index in the 20–40 cm sediment layer. Baoying Lake (BYH), Zhuyu Bay (ZYW), Luyang Lake (LYH), Runyang Wetland (RYSD), Sanwan (SW), Phoenix Island-Islande (YQ), and Jufeng Island (JFD). Values are presented as mean ± standard error (n = 3). Lowercase letters indicate significant differences among wetlands (*p* < 0.05). A stress value of less than 0.2 indicates a good fit of the results.

In terms of archaeal diversity, the archaeal richness and Shannon index in the 0–20 cm and 20–40 cm sediment layers of the LYH wetland were the lowest. In the 0–20 cm sediment layer, the archaeal richness of the BYH and JFD wetlands was significantly higher than that of the other five wetlands (Figure 3a). However, in the 20–40 cm sediment layer, the archaeal richness of the SW, YQ, and JFD wetlands was significantly higher than that of the other four types of wetlands, with the BYH wetland having the second-highest archaeal richness (Figure 3b). Regarding the archaeal Shannon index, the BYH wetland had the highest archaeal Shannon index in the 0–20 cm sediment layer (Figure 3c). In the 20–40 cm sediment layer, the SW wetland had the highest archaeal Shannon index, which was significantly higher than that of the other six wetlands, followed by the BYH, YQ, and JFD wetlands (Figure 3d).

Regarding bacterial diversity, in contrast to archaea, the bacterial richness and Shannon index in the 0–20 cm and 20–40 cm sediment layers of the BYH wetland were the lowest, rather than the LYH wetland. In the 0–20 cm sediment layer, the wetlands with higher bacterial richness were RYSD, SW, and YQ wetlands, while those with intermediate levels were ZYW, LYH, and JFD wetlands (Figure 3e). In the 20–40 cm sediment layer, the JFD wetland had the highest bacterial richness, followed by the SW wetland (Figure 3f). In terms of bacterial Shannon index, there was no significant difference among the seven wetlands in the 0–20 cm sediment layer (Figure 3g). In the 20–40 cm sediment layer, the JFD wetland had the highest bacterial Shannon index, followed by ZYW wetland, and then LYH, SW, and YQ wetlands at the same level, followed by RYSD, and finally BYH wetland (Figure 3h).

**Figure 4:**
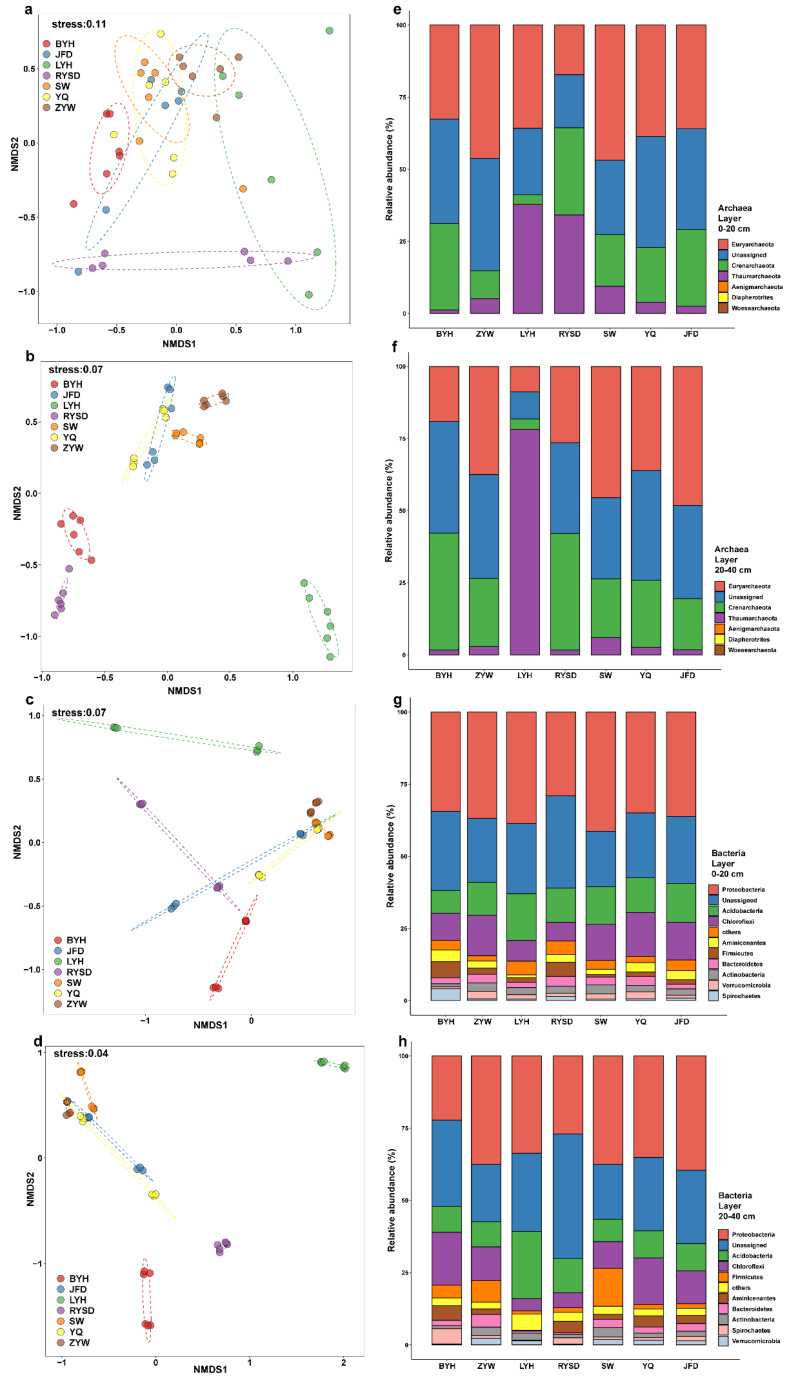
β-diversity analysis and microbial community composition of sediments in different wetlands. (**a**) β-diversity analysis of archaea in the 0–20 cm sediment layer; (**b**) β-diversity analysis of archaea in the 20–40 cm sediment layer; (**c**) β-diversity analysis of bacteria in the 0–20 cm sediment layer; (**d**) β-diversity analysis of bacteria in the 20–40 cm sediment layer; (**e**) Phylum-level composition of archaeal community composition in the 0–20 cm sediment layer; (**f**) Phylum-level composition of archaeal community composition in the 20–40 cm sediment layer; (**g**) Phylum-level composition of bacterial community composition in the 0–20 cm sediment layer; (**h**) Phylum-level composition of bacterial community composition in the 20–40 cm sediment layer.

Through β-diversity analysis of microbial communities, we can observe the distribution and distances of different wetland archaeal and bacterial samples in the plot, thereby assessing the similarities and differences in archaea and bacteria among wetlands. In the β-diversity analysis of archaea in the 0–20 cm sediment layer, the stress value was 0.11. The samples from the RYSD were more concentrated in the NMDS1 and NMDS2 dimensions, indicating the consistency among its internal samples. Moreover, the samples from the BYH and JFD wetlands were closer to each other in the plot, suggesting higher similarity in the characteristics of archaeal relative abundance (Figure 4a). In the analysis of archaeal relative abundance in the 0–20 cm sediment layer, the phylum abundance ratios of BYH and JFD were relatively similar, which was consistent with the results of β-diversity analysis. Meanwhile, the relative abundances of the phyla Aigarchaeota, Diapherotrites, and Woesarchaeota were low in all seven wetlands (Figure 4e). In the NMDS analysis of archaea in the 20–40 cm sediment layer, the stress value was 0.07. The samples from the seven wetlands were more concentrated in the two dimensions, but the samples from the LYH wetland were more distant from the other six wetlands (Figure 4b). In the archaeal relative abundance plot, the archaeal relative abundance of LYH differed from the other six wetlands, with the phylum Thaumarchaeota having the highest proportion in LYH but a relatively low proportion in other wetlands. Notably, the proportion of Thaumarchaeota in the 0–20 cm sediment layer was similar between LYH and RYSD, while in the 20–40 cm sediment layer, the relative abundance of Thaumarchaeota increased in LYH but decreased in RYSD (Figure 4f).

In the NMDS analysis of bacteria in the 0–20 cm sediment layer, the stress value was 0.07, with the samples from the RYSD being more concentrated in the two dimensions (Figure 4c). In the bacterial relative abundance plot, the phylum Spirochaetes had the highest relative abundance in the BYH wetland, while the phylum Proteobacteria had a significant proportion in all seven wetlands (Figure 4g). In the 20–40 cm sediment layer, the stress value of NMDS analysis was 0.04, with the samples from JFD and YQ being closer to each other, while the samples from BYH were more distant from the other six sites (Figure 4d). The bacterial relative abundance showed that the phylum Spirochaetes had the highest relative abundance in the BYH wetland, and the phylum proportions of YQ and JFD were relatively similar. Compared with the 0–20 cm sediment layer, the phylum proportions changed to some extent, but Proteobacteria still had a relatively high proportion (Figure 4h).

**Figure 5:**
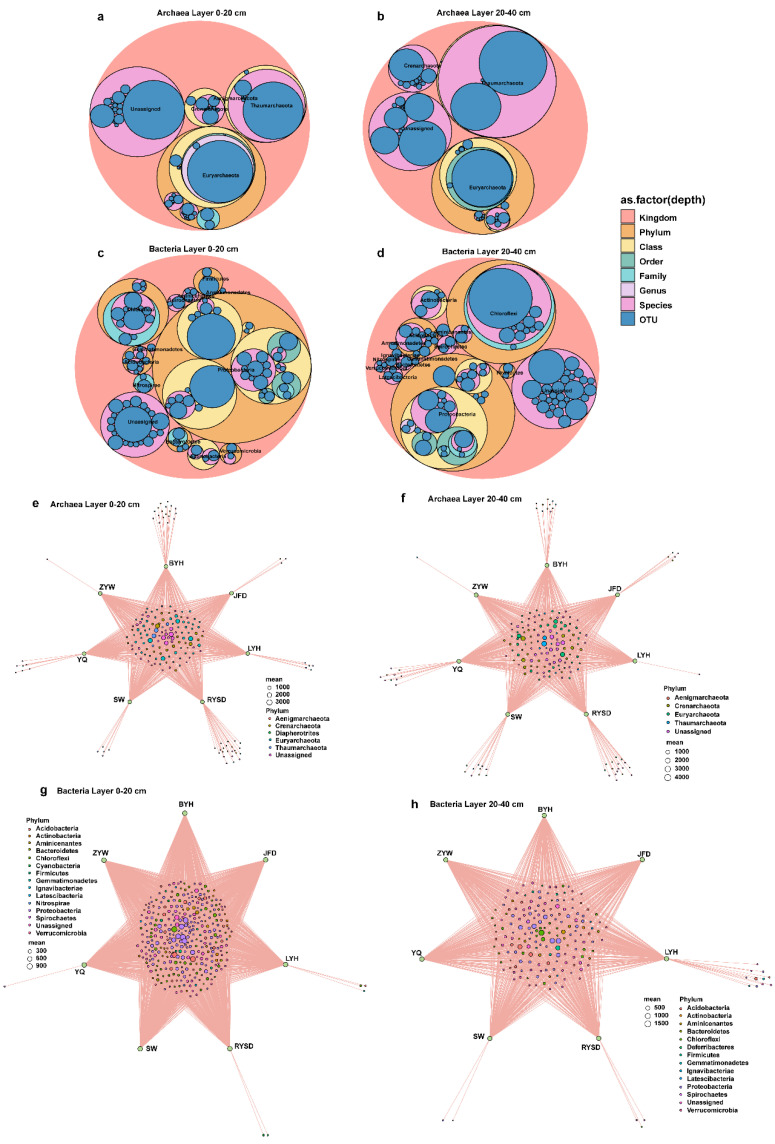
Maptree of microbial communities and bipartite association network relationships of microbial communities. (**a**) Maptree of archaeal community in the 0–20 cm sediment layer; (**b**) Maptree of archaeal community in the 20–40 cm sediment layer; (**c**) Maptree of bacterial community in the 0–20 cm sediment layer; (**d**) Maptree of bacterial community in the 20–40 cm sediment layer; (**e**) Network of archaeal community in the 0–20 cm sediment layer; (**f**) Network of archaeal community in the 20–40 cm sediment layer; (**g**) Network of bacterial community in the 0–20 cm sediment layer; (**h**) Network of bacterial community in the 20–40 cm sediment layer.

To more intuitively display the dominant phyla of archaea and bacteria in different sediment layers, we used Maptree analysis. It was found that in both the 0–20 cm and 20–40 cm sediment layers, the dominant phyla of archaea were Euryarchaeota and Thaumarchaeota, while the dominant phyla of bacteria were Proteobacteria and Chloroflexi (Figure 5a–d). The Venn diagram showed that the core archaeal phyla in the 0–20 cm layer were Crenarchaeota, Diapherotrites, Euryarchaeota, and Thaumarchaeota, and the core bacterial phyla were Acidobacteria and Proteobacteria; in the 20–40 cm layer, the core archaeal phyla were Crenarchaeota, Euryarchaeota, and Thaumarchaeota, the core bacterial phyla included an additional Chloroflexi, and Diapherotrites disappeared from the core phyla (Figure 5e–h).

### 3.3. The Construction of Microbial Neutral Communities in Urban Wetlands

**Figure 6:**
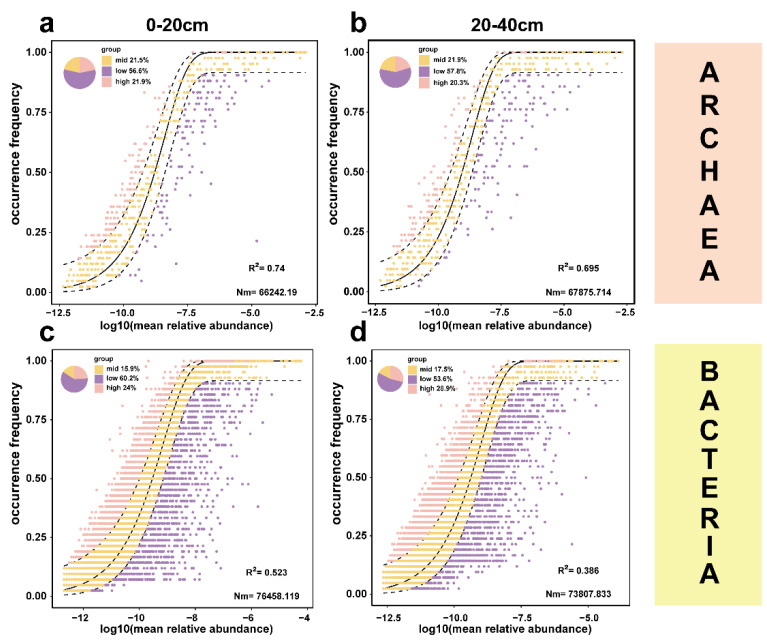
NCM of Microbial Communities. (**a**) Archaea in the 0–20 cm sediment layer; (**b**) Archaea in the 20–40 cm sediment layer; (**c**) Bacteria in the 0–20 cm sediment layer; (**d**) Bacteria in the 20–40 cm sediment layer. In the figure, each data point corresponds to a species. The abscissa denotes the mean relative abundance, while the ordinate represents the frequency of species occurrence. Each panel contains two curves: a solid line and a dashed line. The coefficient of determination (R-squared, Rsq) quantifies the goodness of model fit, where higher values indicate a better correspondence between the neutral model and empirical data. The parameter Nm represents the migration–dispersal magnitude, and a higher Nm value suggests a more homogeneous species distribution across communities.

We constructed a neutral model to explore the assembly processes of the microbiome. The results showed that the archaeal community in the 0–20 cm sediment layer had the highest fitting coefficient with an R^2^ value of 0.74 and a migration rate Nm of 66,242.19 (Figure 6a). For the 20–40 cm sediment layer, the fitting coefficient of archaea was 0.695, slightly lower than that in the 0–20 cm sediment layer, and the migration rate Nm was 67,875.714 (Figure 6b). The fitting results of archaea in these two sediment layers indicated that stochastic processes contributed more to the assembly of archaeal communities, and the impact of stochastic processes was more obvious in the 0–20 cm sediment layer than in the 20–40 cm layer. In terms of bacteria, the R^2^ values in the 0–20 cm and 20–40 cm sediment layers were 0.523 and 0.385, and the migration rates Nm were 76,458.119 and 73,807.833, respectively (Figure 6c,d). This indicated that with the increase in sediment depth, the influence of stochastic processes on the bacterial community gradually weakened. In addition, the R^2^ values of bacteria in different sediment layers were lower than those of archaea in the corresponding layers, indicating that the neutral process had a certain explanatory power for bacteria, but it was weaker than that for archaea. The Nm values in the corresponding sediment layers were higher than those of the archaeal community, suggesting that the distribution of the bacterial community was more uniform than that of the archaeal community.

### 3.4. The Impact of Physicochemical Properties on Sediment Microbial Communities

**Figure 7:**
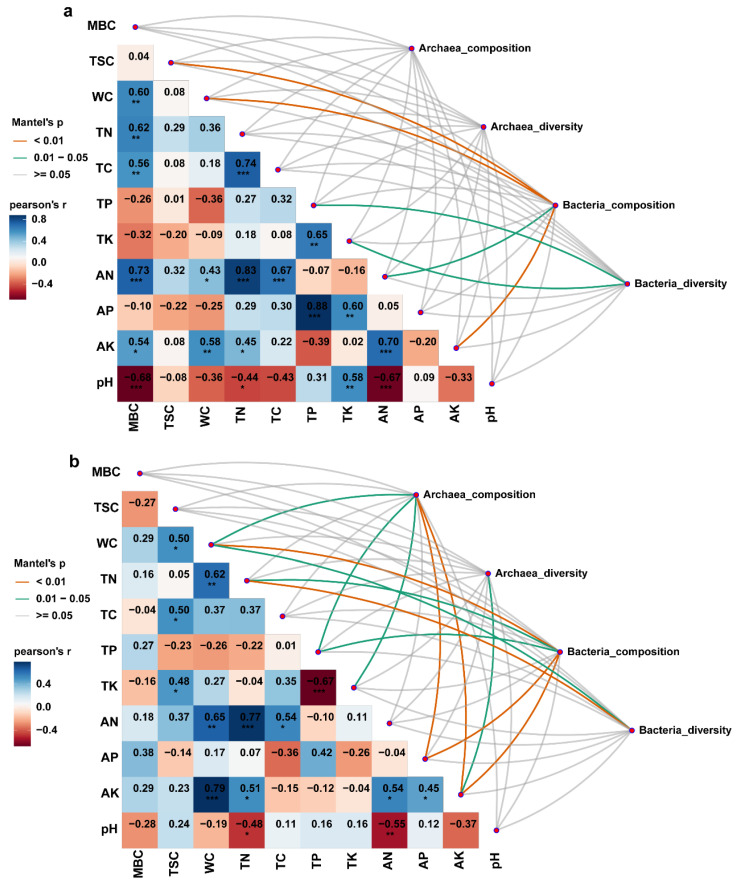
Correlation between sediment physicochemical properties and microbial communities (n = 3). (**a**) 0–20 cm sediment layer; (**b**) 20–40 cm sediment layer. The width of the lines is proportional to the partial Mantel statistics, and the color of the lines indicates statistical significance (orange, *p* < 0.01; green, 0.01 < *p* < 0.05; gray, *p* ≥ 0.05). *, significant at the 0.05 level; **, significant at the 0.01 level; ***, significant at the 0.001 level. Pairwise comparisons of sediment physicochemical factors are also shown, with the color gradient representing the Pearson correlation coefficient.

To investigate the key environmental factors influencing microbial community composition and diversity, we employed the Mantel test to analyze the correlation between microbial communities and environmental variables across different sediment layers. In the 0–20 cm sediment layer, the results indicated that the composition and diversity of archaeal communities were not significantly affected by environmental factors. In contrast, the bacterial community composition was significantly influenced by TSC, WC, AN, and AK, while changes in bacterial diversity were primarily affected by TP and TK (Figure 7a). In the 20–40 cm sediment layer, the impact of environmental factors on microbial communities was more complex. For archaeal communities, WC, TP, TK, AP, and AK significantly affected their composition, whereas archaeal diversity was only significantly influenced by AK. For bacterial communities, their composition was significantly affected by WC, TN, TP, AP, and AK, while bacterial diversity was significantly regulated by WC and TN (Figure 7b). Overall, by integrating the results of the Mantel test from both sediment layers, we found that microbial community composition was significantly more influenced by environmental factors than community diversity.

### 3.5. Analysis of the Potential Functions of Sediment Microbes in Urban Wetlands

**Figure 8:**
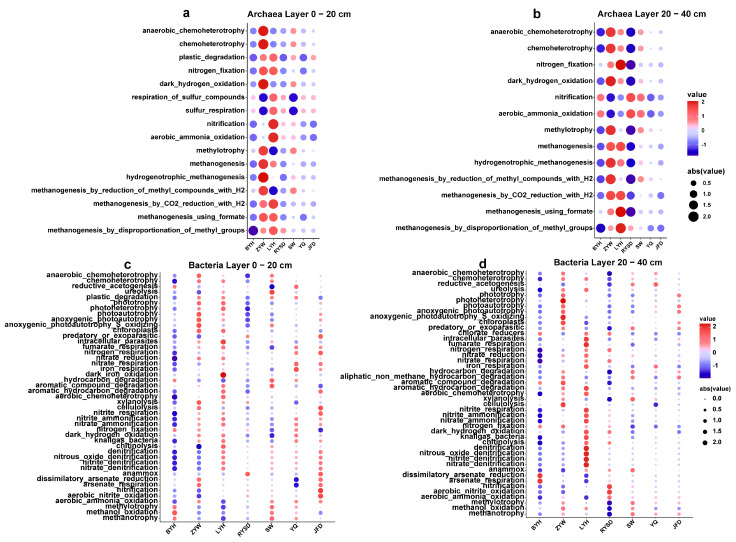
Relative abundance of potential functional categories obtained using Microeco. (**a**) Potential functions of archaea in the 0–20 cm sediment layer; (**b**) Potential functions of archaea in the 20–40 cm sediment layer; (**c**) Potential functions of bacteria in the 0–20 cm sediment layer; (**d**) Potential functions of bacteria in the 20–40 cm sediment layer.

By conducting functional prediction analyses of archaea and bacteria in two sediment layers (0–20 cm and 20–40 cm) across seven wetlands, we elucidated the distribution characteristics and variation patterns of functional genes in microbial communities among different wetlands. For archaea, in the 0–20 cm sediment layer, the majority of functional genes were predominantly concentrated in the ZYW and LYH wetlands. Specifically, the abundance of carbon cycling-related functional genes was relatively high in the ZYW wetland, which also exhibited robust chemoheterotrophic activity. The LYH wetland showed a high abundance of carbon cycling-related functional genes, and notably, the genes associated with nitrification and aerobic ammonia oxidation were exclusively abundant in the LYH wetland (Figure 8a). In contrast, the distribution of functional genes in the 20–40 cm sediment layer exhibited minimal changes. The ZYW wetland maintained a high level of carbon cycling-related functional genes, while the abundance of nitrification and aerobic ammonia oxidation genes in the LYH wetland decreased. Conversely, these genes significantly increased in the RYSD and SW wetlands. Moreover, apart from the aforementioned two functional genes, the overall abundance of other functional genes in the LYH wetland increased (Figure 8b).

For bacteria, in the 0––20 cm sediment layer, the majority of functional genes were highly abundant in the ZYW, LYH, and JFD wetlands. However, functional genes related to carbon cycling were primarily concentrated in the BYH and SW wetlands. The ZYW wetland exhibited high abundance of functional genes associated with light utilization, while functional genes related to nitrogen cycling were highly abundant in the LYH and JFD wetlands (Figure 8c). In the 20–40 cm sediment layer, the ZYW and LYH wetlands maintained high levels of functional gene abundance. The ZYW wetland further enhanced its functional genes related to light utilization, and the LYH wetland showed an improved performance in nitrogen cycling genes. However, the abundance of nitrogen cycling-related genes in the JFD wetland significantly decreased (Figure 8d). In summary, significant differences exist in the distribution of archaeal and bacterial functional genes among different wetlands and sediment layers. The ZYW wetland exhibited superior performance in carbon cycling and light utilization functional genes, while the LYH wetland possessed unique advantages in genes related to carbon and nitrogen cycling. Additionally, the distribution of functional genes exhibited certain dynamic changes with increasing sediment depth.

## 4. Discussion

### 4.1. Different Types of Wetlands and Their Ecosystem Efficiency

Sediments are important carbon pools in wetland ecosystems, undergoing slow carbon exchange with the atmosphere. When carbon in sediments is stably preserved, it will not be released into the atmosphere in the form of carbon dioxide, thus playing a carbon sink role in the global carbon cycle [32]. The total carbon content of the LYH wetland is the highest among all wetlands, indicating its strong carbon storage capacity. In addition, the pH values of the two sediment layers in the LYH wetland are significantly lower than those in other wetlands, showing weak acidity, which may be affected by the surrounding drainage and irrigation stations. A lower pH value may cause the surfaces of metal oxides and hydroxides (such as iron and aluminum oxides and hydroxides) to carry positive charges, which is conducive to the adsorption of organic carbon [33,34], thereby enhancing its carbon sink function. The nitrogen content of the LYH wetland is also relatively high, and nitrogen is a key element for maintaining the balance of aquatic ecosystems [3]. The supply level of nitrogen directly affects the growth of aquatic plants, and its transformation in water helps maintain the acid–base balance of the water body. The formation, degradation, and release of nitrogen are mainly controlled by the environmental conditions and dynamic factors of organic matter mineralization sediments [35].

In contrast, the functional genes related to the carbon cycle in the ZYW wetland remain at a high level, and we speculate that the ZYW wetland may have strong capabilities in photoheterotrophy and anaerobic degradation processes. RYSD shows prominent characteristics in the phosphorus cycle. Studies have shown that the phosphorus content in the middle and lower reaches of the Yangtze River is relatively high [36]. We guess that RYSD may be closer to the middle and lower reaches of the Yangtze River, thus leading to the high phosphorus environment in the RYSD. The high phosphorus environment may have shaped its unique bacterial community structure, and the efficient phosphorus utilization ability of bacteria has further maintained the phosphorus cycle efficiency of this wetland. Understanding the differences in physical and chemical properties between different wetlands provides an important basis for in-depth research on the mechanisms of wetlands’ role in carbon and nitrogen cycles.

### 4.2. Impact of Physical and Chemical Properties on Microbial Community Assembly

Wetlands have important ecological benefits in global ecosystems, and their functions and health status are comprehensively affected by various factors such as sediment physical and chemical properties and microbial communities. There is an inherent connection between microbial communities and the physical and chemical properties of sediments. Previous studies have confirmed that archaea and bacteria usually show high abundance under high pH conditions [37,38]. However, in this study, the bacterial composition and abundance in the BYH wetland showed an opposite trend under high pH conditions, indicating that the response of microbial communities to pH may vary depending on wetland types and specific environmental backgrounds. In addition, the impact of nitrogen content on microbial communities has also attracted our attention. Microorganisms can use nitrogen sources such as ammonia, nitrate, nitrite, or amino acids for growth [39,40,41,42,43,44,45]. Based on these findings, we hypothesize that nitrogen content may affect the structure and function of archaeal and bacterial communities to a certain extent. Carbon is also a key determinant of the structure and function of sediment microbial communities [46]. The diversity of microbial catabolism is affected by organic carbon storage [47]. In our study, the bacterial diversity in the 20–40 cm sediment layer was significantly affected by TSC, which is consistent with previous research results, further confirming the important regulatory role of sediment carbon storage on microbial communities. In our research results, phosphorus and potassium showed significant correlations with microbial communities. Phosphorus can change the structure of bacterial communities but does not affect their species richness [48]. Potassium regulates the physiological functions of microbial communities by affecting their molecular homeostasis, because the physiological functions of many bacteria depend on the normal operation of K⁺ channels [49]. It is worth noting that in the two sediment layers, bacteria have a wider correlation with soil physical and chemical properties.

### 4.3. Stochastic Processes and Deterministic Processes (Based on Neutral Community Model)

Similarly to most studies, this study clarifies the association between certain physical and chemical characteristics and microbial functions, but the research focuses on different wetlands are different. For example, studies on the Baiyangdian wetland mainly focus on the analysis of nitrogen and phosphorus elements [50]; other studies conduct ecological risk assessments by analyzing heavy metals in sediments [51]. In contrast, on the basis of exploring the association between physical and chemical characteristics and microbial functions, this study further reveals the ecological processes of microbial communities in different sediment layers of Yangzhou wetlands with the help of the neutral community model.

The results show that the archaeal communities in Yangzhou wetlands are highly affected by stochastic processes, which is similar to the study by Gao et al. in coastal wetland [52], while the bacterial communities are mainly driven by deterministic processes, which is similar to the view of Hu et al. [53]. Specifically, the R^2^ values of archaea in the 0–20 cm and 20–40 cm layers are 0.74 and 0.695, respectively, indicating a high degree of fit with the neutral model, while the R^2^ values of bacteria (0.523 and 0.385) are low, indicating that stochastic processes have a weak impact on them. These findings clarify the characteristics of core community composition and indicate that the universal existence of stochastic processes may mean stronger ecological stability when facing disturbances, especially in deeper sediment layers. This dynamic is worthy of further exploration on a spatiotemporal gradient. However, our study only compares the differences in the assembly of archaeal and bacterial communities in Yangzhou wetlands, and does not analyze the combined impact of stochastic and deterministic processes on the assembly process of microbial communities [54].

Combined with β diversity analysis, it can be inferred that in the NMDS diagram, archaeal samples show a high degree of aggregation, which reflects the community similarity dominated by random diffusion; in contrast, bacterial samples are more discretely distributed in different wetlands, which may be the result of community differentiation caused by environmental filtering. Relevant studies have shown that WC, nitrogen, and potassium are the deterministic factors shaping bacterial communities [55,56]. In our correlation research results, the bacterial community has a strong association with WC, TN, and AK.

### 4.4. Key Microbial Taxa and Their Ecological Functions

Studies have shown that Thaumarchaeota play a key role in carbon and nitrogen cycles [57,58]. Although the overall abundance of archaea in the LYH wetland is relatively low, the relative abundance of Thaumarchaeota in it is higher than that in other wetlands. We believe that the higher abundance of Thaumarchaeota is the reason why the LYH wetland has a strong functional response to carbon and nitrogen functional genes. Ammonia-oxidizing archaea (AOA) in Thaumarchaeota have a high affinity for ammonia oxidation and can efficiently utilize energy through aerobic carbon fixation pathways [59]. They are important primary producers in various environments, including terrestrial ecosystems [60], shallow-water layers [61,62], deep-water layers [63,64], deep-sea sediments [65], and the deepest trench—the Mariana Trench [47].

In addition, the relative abundance of Acidobacteria in the LYH wetland is also relatively high. Acidobacteria is a bacterial phylum widely distributed in soil and sediments, and most of its members can obtain nitrogen from mineral and organic sources [66]. Moreover, the genome of Acidobacteria contains genes encoding enzymes that degrade complex carbohydrate polymers [67,68]. Acidobacteria usually act as decomposers in soil and play an important role in the organic matter cycle through their potential to degrade polymeric carbon compounds [69]. Although the importance of Thaumarchaeota and Acidobacteria in carbon and nitrogen cycles has been fully confirmed, the interaction mechanism between them and other microorganisms is still unclear. In-depth study of these interactions will help us more comprehensively understand the function and stability of the LYH wetland ecosystem, so as to provide help for better construction and management of wetland ecosystems.

### 4.5. Practical Applications and Technical Improvement Suggestions

In conclusion, the burial and deposition of sediments are the results of the combined action of various environmental factors [35]. Our study reveals the commonalities and differences between urban wetlands, identifies the important roles of Thaumarchaeota and Acidobacteria in sediments, and predicts the ecological functions of each wetland, laying a foundation for future in-depth research on wetland ecosystems. However, wetland ecological functions are also affected by factors such as heavy metals, wetland plants, and anthropogenic pollution. For example, heavy metal cadmium will affect the nitrogen cycle of wetlands [70], and the absorption and transformation of metal elements by wetland plants will affect the enrichment of metal element content in wetlands [71]. This study did not measure the content of heavy metals in sediments, wetland plants, and anthropogenic pollution factors, which is a potential limitation. In the future, we will start from these aspects to understand the characteristics of Yangzhou wetlands at a deeper level and further analyze the driving mechanism of wetland microbial community assembly. These improvements will provide more targeted theoretical support for wetland protection and management.

## 5. Conclusions

This study analyzed different sediment layers of seven urban wetlands in Yangzhou and found that the LYH wetland has high carbon and nitrogen contents with weakly acidic pH, while the RYSD and YQ wetlands have high phosphorus contents. It was also discovered that the LYH wetland has potential advantages in carbon and nitrogen cycling functions, and the ZYW wetland has potential advantages in carbon cycling and light utilization functions. Thaumarchaeota and Euryarchaeota are the dominant archaea, while Proteobacteria and Acidobacteria are the dominant bacteria. Their abundance of proportions in the microbial community affects the potential functions of the wetlands. In addition, the archaeal community is mainly dominated by stochastic processes, while the bacterial community is mainly regulated by deterministic processes. These research results preliminarily clarify the relationships between the physicochemical properties of sediments, microbial communities, and ecological functions in Yangzhou urban wetlands, providing an important reference for understanding the functional diversity of plain wetland ecosystems. In the future, based on this research, heavy-metal-pollution indicators will be collected to further analyze the physicochemical properties of wetland sediments, and null models will be combined to gain a deeper understanding of the construction of wetland microbial communities.

## Data Availability

The raw data supporting the conclusions of this article will be made available by the authors on request.

## Supplementary Materials

The following supporting information can be downloaded at: https://www.mdpi.com/article/10.3390/microorganisms13081843/s1, Figure S1: The change in species richness of different samples under different sequencing depths.
