# Supplementary material for: Urban Wetland Sediments in Yangzhou: Physicochemical Properties, Microbial Communities, and Functional Associations
Source: Microorganisms. 2025 Aug 7;13(8):1843. doi: 10.3390/microorganisms13081843 (PMC12388665; doi:10.3390/microorganisms13081843)
Supplement: Supplementary file 1 [file microorganisms-13-01843-s001.zip › microorganisms-3780509-supplementary.pdf]

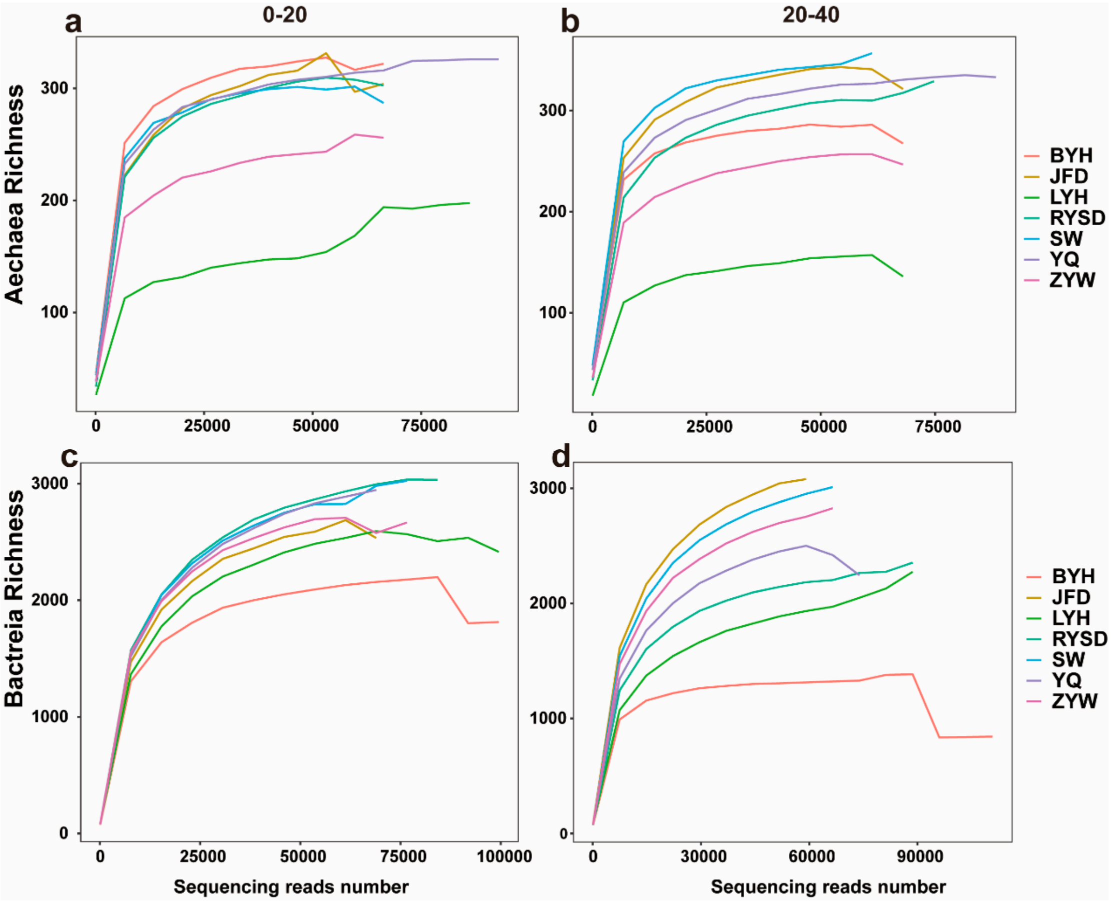

FigureS1 The change in species richness of different samples under different sequencing depths. (a) Archaeal richness in the 0–20 cm sediment layer; (b) Archaeal richness in the 20–40 cm sediment layer; (c) Bacterial richness in the 0–20 cm sediment layer; (d) Bacterial richness in the 20–40 cm sediment layer. Baoying Lake (BYH), Zhuyu Bay (ZYW), Luyang Lake (LYH), Runyang Wetland (RYSD), Sanwan Wetland (SW), Phoenix Island-Islande (YQ), andJufeng Island (JFD).
